# Foamy Virus Vectors Transduce Visceral Organs and Hippocampal Structures following *In Vivo* Delivery to Neonatal Mice

**DOI:** 10.1016/j.omtn.2018.07.006

**Published:** 2018-08-03

**Authors:** John R. Counsell, Rajvinder Karda, Juan Antinao Diaz, Louise Carey, Tatiana Wiktorowicz, Suzanne M.K. Buckley, Shima Ameri, Joanne Ng, Julien Baruteau, Filipa Almeida, Rohan de Silva, Roberto Simone, Eleonora Lugarà, Gabriele Lignani, Dirk Lindemann, Axel Rethwilm, Ahad A. Rahim, Simon N. Waddington, Steven J. Howe

**Affiliations:** 1 Gene Transfer Technology Group, EGA Institute for Women’s Health, University College London, London WC1E 6HX, UK; 2 Dubowitz Neuromuscular Centre, Molecular Neurosciences Section, Developmental Neurosciences Programme, UCL Great Ormond Street Institute of Child Health, 30 Guilford Street, London WC1N 1EH, UK; 3 NIHR Great Ormond Street Hospital Biomedical Research Centre, 30 Guilford Street, London WC1N 1EH, UK; 4 Department of Pharmacology, UCL School of Pharmacy, University College London, London WC1N 1AX, UK; 5 Universität Würzburg, Institut für Virologie und Immunbiologie, Versbacher Str. 7, 97078 Würzburg, Germany; 6 Reta Lila Weston Institute and Department of Molecular Neuroscience, UCL Institute of Neurology, London WC1N 3BG, UK; 7 Department of Clinical and Experimental Epilepsy, Queen Square House, UCL Institute of Neurology, London WC1N 3BG, UK; 8 Institute of Virology, Technische Universität Dresden, Dresden, Germany; 9 Center for Regenerative Therapies Dresden (CRTD), Technische Universität Dresden, Dresden, Germany; 10 Wits/SAMRC Antiviral Gene Therapy Research Unit, Faculty of Health Sciences, University of the Witwatersrand, Johannesburg, South Africa

**Keywords:** foamy virus, spumavirus, viral vector, gene therapy, vector tropism, bioimaging, hippocampus

## Abstract

Viral vectors are rapidly being developed for a range of applications in research and gene therapy. Prototype foamy virus (PFV) vectors have been described for gene therapy, although their use has mainly been restricted to *ex vivo* stem cell modification. Here we report direct *in vivo* transgene delivery with PFV vectors carrying reporter gene constructs. In our investigations, systemic PFV vector delivery to neonatal mice gave transgene expression in the heart, xiphisternum, liver, pancreas, and gut, whereas intracranial administration produced brain expression until animals were euthanized 49 days post-transduction. Immunostaining and confocal microscopy analysis of injected brains showed that transgene expression was highly localized to hippocampal architecture despite vector delivery being administered to the lateral ventricle. This was compared with intracranial biodistribution of lentiviral vectors and adeno-associated virus vectors, which gave a broad, non-specific spread through the neonatal mouse brain without regional localization, even when administered at lower copy numbers. Our work demonstrates that PFV can be used for neonatal gene delivery with an intracranial expression profile that localizes to hippocampal neurons, potentially because of the mitotic status of the targeted cells, which could be of use for research applications and gene therapy of neurological disorders.

## Introduction

Since the mid-1980s, recombinant DNA technology and the nascent field of viral vectorology have been developed as therapeutic tools for the treatment of inherited genetic diseases.^1^, ^2^, ^3^, ^4^, ^5^, ^6^ Many viral vector systems have been explored, with each providing a unique set of characteristics that can be exploited for a specific purpose.^7^

Two of the most widely used viral vectors in research and clinical gene therapy are based on lentiviruses (LVs) and adeno-associated viruses (AAVs). LV vectors have been widely used for their ability to integrate into target cell genomes, which has been key to their recent success in stem cell gene therapy.^8^, ^9^, ^10^, ^11^, ^12^ AAV vectors have shown burgeoning potential for *in vivo* gene therapy, demonstrating safe and efficient transduction of the human brain by intracranial administration.^13^ Furthermore, systemic AAV delivery can deliver body-wide gene expression in humans^14^, ^15^ and many animal models, with some vector serotypes able to cross the blood-brain barrier.^16^, ^17^

Notwithstanding the recent successes of AAVs and LVs, these vectors suffer drawbacks that mitigate continued exploration of alternative systems. AAVs are restricted to transgene payloads below 5.2 kb,^18^, ^19^, ^20^ which restricts fine-tuning of therapeutic cassettes with additional regulatory genetic sequences, such as promoters, enhancers, and microRNA recognition sequences. Additionally, AAV vectors do not commonly integrate into target cell genomes; thus, vector copies are lost after repeated cell divisions.^21^ LV vectors can package considerably larger genes than AAVs and efficiently integrate their DNA into host chromosomes, which has made them a popular choice for stem cell therapy.^22^, ^23^, ^24^ However, like all retroviruses, LV vectors are confounded by the risk of insertional mutagenesis, which is a concern in clinical translation.^25^ In a recent clinical trial for β-thalassemia stem cell therapy, LV vector integration into the HMGA2 proto-oncogene led to transcription of a truncated mRNA and benign clonal dominance.^26^

Foamy virus (FV) vectors are derived from a subfamily of retroviruses known as Spumaretroviridae, possessing several distinguishing properties that show potential for gene therapy. Like AAVs, wild-type FV infections are not associated with pathology.^27^ Prototypic FV (PFV) vectors are derived from an FV strain isolated from a human, although sequence analysis of PFV indicates that it is a chimpanzee isolate.^28^, ^29^, ^30^ PFV vectors have several distinguishing properties that could be exploited for gene therapy, such as a large packaging capacity that can accommodate payloads greater than 9 kb,^31^ a reverse transcription pathway that occurs before target cell entry,^32^, ^33^ dormancy of the preintegration complex in quiescent cells,^34^ and very limited seroprevalence in humans.^35^ PFV vectors have been developed for gene transfer, showing broad cellular tropism that is ascribed to their use of heparin sulfate glycosaminoglycan as a means of cell entry.^36^, ^37^ They are particularly effective at transducing stem cells, showing promise for *ex vivo* treatment of inherited diseases.^38^, ^39^, ^40^, ^41^, ^42^, ^43^ As with other retroviral vectors, PFV vector proviruses integrate into the host genome as part of normal transduction. It has been suggested that PFV vectors may even have a safer integration profile than LV vectors and murine leukemia virus (MLV) retroviral vectors because they tend to integrate outside of active transcription units.^44^

The use of PFV vectors *in vivo* has recently been demonstrated in gene transfer to the regenerating limb tissue of salamanders and for transduction of juvenile pig liver by hydrodynamic injection.^45^, ^46^ Additionally, PFV vectors have been used to deliver their genomic RNA as mRNA *in* vivo.^47^ However, broader use of PFV vectors for permanent *in vivo* transgene delivery remains largely unexplored in mammals.

Here we investigated the *in vivo* transduction characteristics of PFV vectors by delivering transgenes to neonatal mice via intracranial, intravenous, intraperitoneal, and subcutaneous routes of administration. Systemic PFV vector delivery gave expression in a range of visceral organs, whereas intracranial administration gave a region-specific expression profile localized to hippocampal architecture. This hippocampal expression pattern was not observed in mice that received intracranial LV and AAV vectors via the same route of delivery, even when administered at lower doses than PFV vectors. Thus, our data introduce PFV vectors as unique gene transfer agents for use in research and gene therapy, and their discrete brain expression profile provides a novel approach for accurate manipulation of brain function.

## Results

PFV vectors were packaged with either EGFP or luciferase (Luc) driven by the cytomegalovirus (CMV) promoter. PFV-EGFP and PFV-luciferase vectors were delivered to neonatal outbred CD1 mice by intraperitoneal (i.p.), intravenous (i.v.), subcutaneous (s.c.), and intracranial (i.c.) injection on the day of birth.

### Intravenous PFV Administration to Neonatal Mice Produces Expression in Visceral Organs

**Figure 1:**
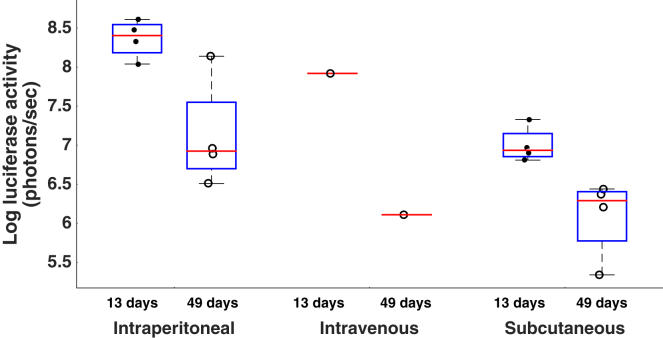
Whole-Body Luciferase Activity Detected in Mice Injected with PFV-CMV-Luciferase by Multiple Routes of Delivery Neonatal mice were injected on the day of birth either intraperitoneally (n = 4), intravenously (n = 1), or subcutaneously (n = 4) and subsequently imaged 13 days and 49 days post-injection to track body-wide luciferase expression. The mean luciferase activity detected in each animal is plotted as an individual dot plot (black dots, day 13 values; white circles, day 49 values) with overlaid boxplots representing 75% confidence intervals of the dotplot distribution (blue boxes) and median lines (red lines). Data show that intraperitoneally delivered luciferase expression remained on day 49 despite expression falling from day 13 (p = 0.0433 by Kruskal-Wallis test). Consistent with this, subcutaneously delivered luciferase was present on day 49 despite a fall in expression from day 13 (p = 0.0209 by Kruskal-Wallis test). Data for the single mouse receiving intravenous injection are shown.

Whole-body bioluminescent images were captured and quantified 13 days and 49 days after neonatal intraperitoneal, intravenous, and subcutaneous administration of PFV-Luc (Figure 1). For intraperitonealy-treated animals, the mean total flux was calculated as 2.6 ± 1.3 × 10^8^ photons/s 13 days post-injection. Expression from intraperitonealy-injected animals remained detectable 49 days post-injection, at which point mean flux was detected at 4.0 ± 6.6 × 10^7^. Subcutaneously-injected animals gave a mean flux of 1.1 ± 0.7 × 10^7^ on day 13 and 1.7 ± 1.1 × 10^6^ on day 49. Intravenous injection was poorly tolerated; three of the four PFV-luciferase cohorts died before the first imaging time point. The remaining animal gave a mean luciferase reading of 8.4 × 10^7^ on day 13, which was detected at 1.3 × 10^6^ on day 49.

**Figure 2:**
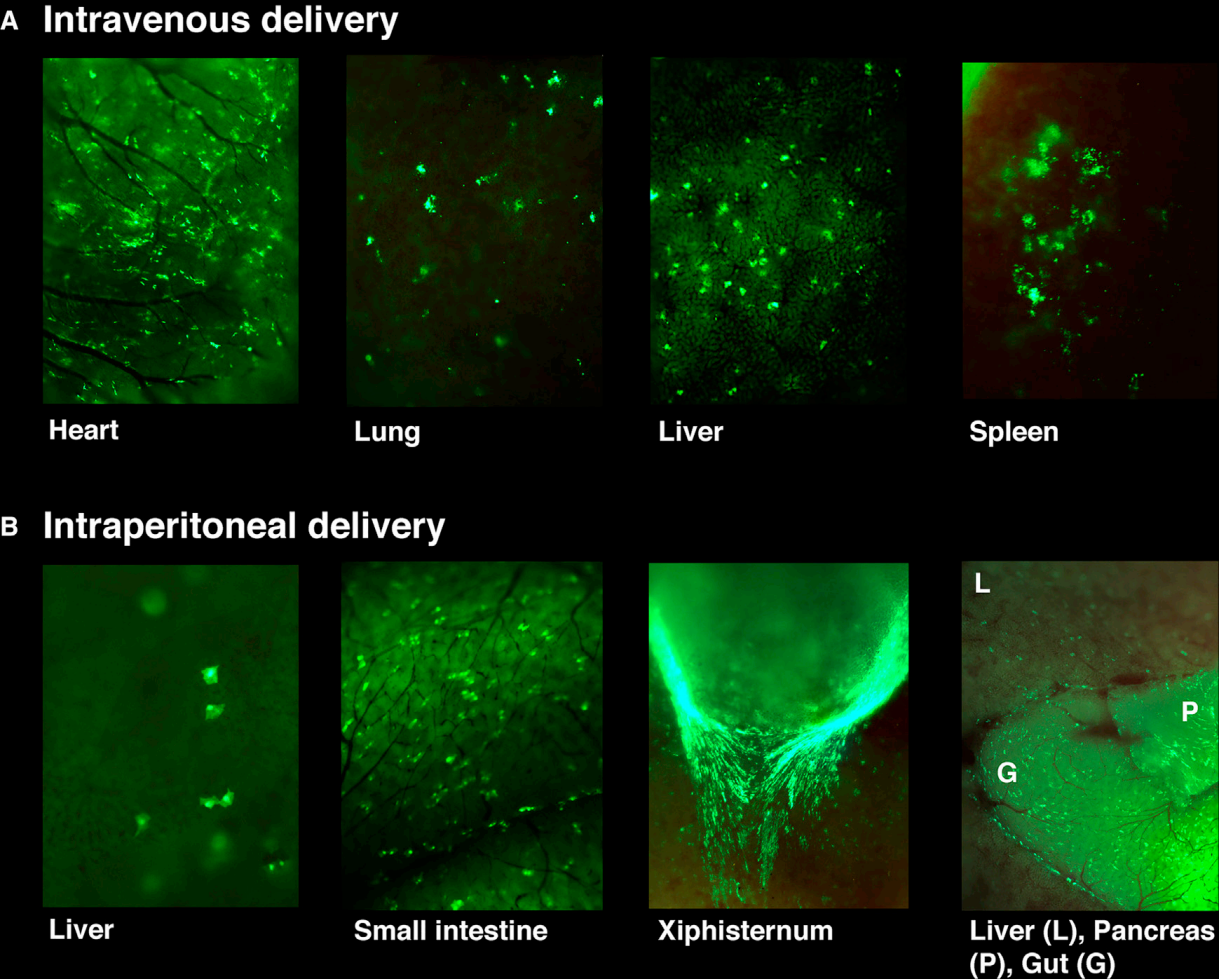
EGFP Fluorescence Imaging of Mice Receiving Systemic Injections of PFV-CMV-EGFP Stereoscopic imaging on day 11 post-injection revealed EGFP expression in transduced visceral organs following intravenous and intraperitoneal vector delivery. (A) Intravenous PFV injection demonstrates EGFP fluorescence in the heart, lung, liver, and spleen (all 20× magnification). (B) Intraperitoneal vector injection gave EGFP fluorescence in the liver (20× magnification), small intestine (30× magnification), xiphisternum (30× magnification), and liver, pancreas, and gut (10× magnification).

PFV-EGFP-injected animals were dissected after 11 days to investigate tissue tropism in greater detail (Figure 2). Intravenous delivery gave expression in the heart, liver, lung, and spleen, whereas intraperitoneal injection transduced the xiphisternum, liver, pancreas, and gut.

### Intracranial PFV Administration to Neonatal Mice Gives Discrete Hippocampal Transgene Expression that Is Not Seen with LV and AAV Vector Technologies

**Figure 3:**
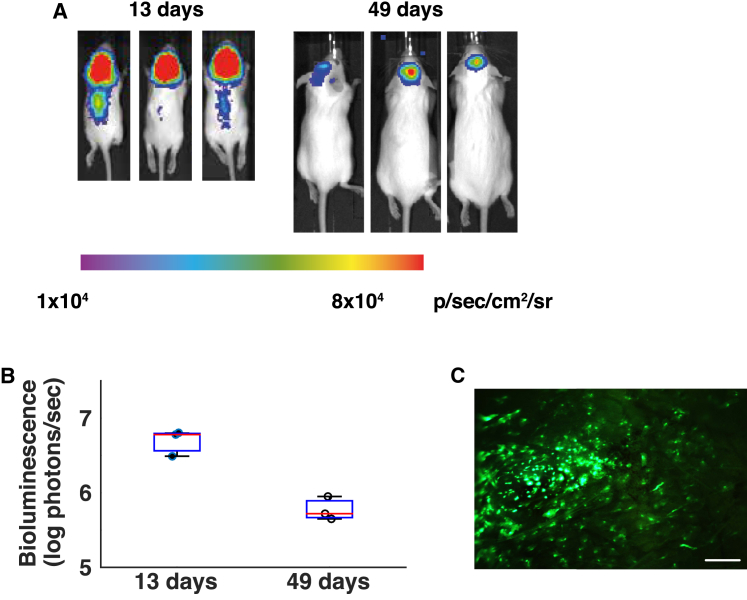
Transgene Expression in the Mouse Brain 13 Days after Neonatal Intracranial PFV Injections (A) Bioluminescent imaging shows expression in the brain, and some in the spinal cord, following intracerebroventricular vector administration on post-natal day 1. Images taken 49 days post-injection show expression limited to the brain. (B) Luciferase expression remained detectable until sacrifice at 49 days but was significantly reduced compared with expression at 13 days (p = 0.0495 by Kruskal-Wallis test). The mean luciferase activity detected in each animal is plotted as an individual dot plot (black dots, day 13 values; white circles, day 49 values), with overlaid box plots representing 75% confidence intervals of the dot plot distribution (blue boxes) and median lines (red lines). (C) Brain EGFP fluorescence was detected through the skull, to the left of the bregma, before subsequent sectioning. Scale bar, 1.25 mm.

Neonatal mice received a unilateral intracranial injection of PFV-luciferase, with bioluminescence quantified at two time points (Figure 3A). 11 days post-injection, we detected luciferase expression in the brains of all animals, with some expression detectable in the spinal cord (mean value of 5.1 ± 1.8 × 10^6^ photons/s). Subsequent imaging 49 days post-injection showed that luciferase expression remained in the brain but was no longer detectable in the spinal cord (mean value of 6.2 ± 2.3 × 10^5^ photons/s). Brain expression produced 8.2-fold less bioluminescence signal at the later time point (p = 0.0495 by Kruskal-Wallis test) (Figure 3B).

We suspected that the higher expression level on day 11 may have been produced by transient expression from plasmid DNA carried over from PFV vector production. We investigated this by injecting 1.5 μg (equivalent to total plasmid used to transfect a whole 10-cm dish of producer cells) of naked plasmid DNA expressing a luciferase expression cassette, but the resulting bioluminescence was indiscernible from uninjected controls 11 days post-injection (Figure S3).

**Figure 4:**
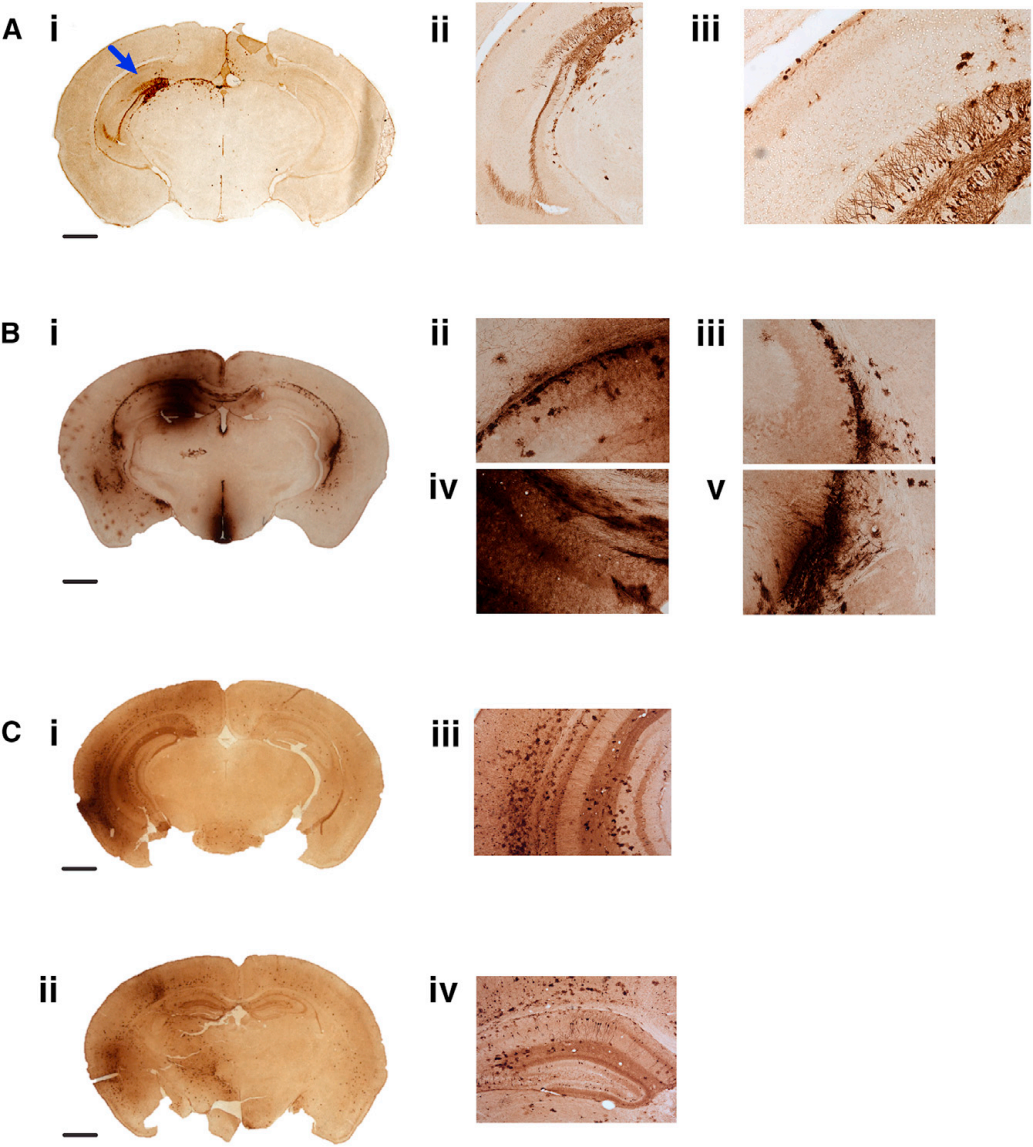
Intracranial Biodistribution of PFV-EGFP, LV-EGFP, and AAV-EGFP Vectors 11 Days after Intracranial Injection into Neonatal Mice (A) Administration of 2.5 × 10^7^ PFV-EGFP vector genome copies to the lateral ventricle gives EGFP immunostaining localized to hippocampal cells (indicated by the blue arrow). Scale bar, 5 μm (i); 20× magnification (ii); 40× magnification (iii). (B) Injection of 4.2 × 10^6^ LV-EGFP genome copies by the same route of delivery gives staining in a variety of brain regions with extensive spread from the cerebral ventricles. Scale bar, 5 μm (i); 40× magnification (ii–v). (C) A dose of 2.5 × 10^6^ AAV-EGFP genome copies also shows indiscriminate spread of vector expression, with signs of migration from the ventricles, indicating that even low copy numbers of AAVs do not demonstrate site-specific localization. Scale bars, 5 μm (i and ii); 40× magnification (iii and iv).

To investigate the intracranial localization of PFV expression, we administered PFV-EGFP to the brains of newborn mice on post-natal day 1, with unilateral intracranial injections aimed toward the anterior horn of the lateral ventricle on the left side of the brain. Post-mortem analysis 11 days post-injection showed EGFP fluorescence through the top of the skull to the left of bregma, which was the approximate site of injection (Figure 3C). Dissected brains were further analyzed by immunostaining to investigate intracranial PFV biodistribution (Figures S1A and S2). Focused evaluation of the stained brain regions revealed that EGFP expression was localized to the hippocampus (Figure 4A). Further magnification revealed dense expression localized to the dentate gyrus, with staining detected throughout the associated architecture.

LV and AAV vectors are commonly used for brain gene therapy, having demonstrated widespread transduction of the mouse brain in a variety of applications.^16^, ^17^, ^48^, ^49^, ^50^ Thus, we sought to compare the intracranial biodistribution of PFV-EGFP vectors with AAV and LV vector technologies.

LV-EGFP vectors were delivered at a lower dose (4.2 × 10^6^ genome copies) than PFV-EGFP vectors (2.5 × 10^7^ genome copies), but LV clearly showed greater diffusion from the lateral ventricle injection site, with staining detected in a variety of brain structures (Figure 4B; Figure S1B). However, administration of LV-EGFP to adult mice limited vector diffusion through the brain, with expression restricted to the hippocampus, piriform cortex, and olfactory bulb at lower levels than observed after neonatal injection (Figure S4).

AAV-EGFP was initially delivered to mice at a dose of 2.5 × 10^10^ total vector genomes, producing widespread expression that migrated extensively from the injection site (Figure S1C). However, to account for the 3 orders of magnitude difference between PFV and AAV vector doses, we additionally administered AAV-EGFP at a lower dose (2.5 × 10^6^ vector genome copies) to interrogate the importance of vector genome copy number in the hippocampal localization of PFV-EGFP. Surprisingly, the lower dose of AAV-EGFP continued to exhibit broader expression than PFV-EGFP, suggesting that vector genome copy number alone was not the exclusive reason for intracranial biodistribution (Figure 4C).

To confirm the phenotype of EGFP-expressing cells, brain sections were stained for EGFP and the neuron-specific marker NeuN, with z stacks captured by confocal microscopy. Confocal images showed that PFV-treated sections were localized to neurons of the dentate gyrus, aside from some non-specific labeling of blood vessels because of suboptimal perfusion (Figure S5).

## Discussion

Viral vectors are rapidly being developed as gene therapy agents for an increasing range of diseases. This naturally brings a growing demand for broader functionality and diversity of viral vectors and a need to thoroughly examine novel and existing vector technologies to fully understand their capabilities. Here we have shown that PFV vectors are capable tools for direct *in vivo* gene delivery, particularly in the developing neonatal hippocampus, which adds a new dimension to their role in the growing field of gene therapy.

The ability of PFV vectors to integrate into the host genome makes them an appropriate choice for *ex vivo* gene therapy. PFV vectors have been assessed extensively for *ex vivo* manipulation of hematopoietic stem cells and engraftment in disease models in rodent and canine models.^42^, ^51^ Our study provides a comprehensive examination of their biodistribution and efficacy following *in vivo* gene delivery to neonatal mice.

Our investigations were not only designed to show proof of concept for neonatal gene therapy with PFV vector technology but also to investigate the behavior of these vectors in postnatal tissues for physiological research purposes. It has been reported that PFV vectors absolutely require mitosis for efficient transduction of target cells, but these vectors can form a stable transduction intermediate in quiescent (G0) cells.^34^, ^52^, ^53^ In our investigations, we restricted our studies to newborn mice because we expected that murine organs would retain some degree of mitotic activity during postnatal development.

Systemic injection of PFV vectors gave transgene expression in several visceral organs, with luciferase expression detectable until termination of the experiments 49 days post-injection. However, our imaging data showed that EGFP-positive cells were sparsely distributed throughout the transduced organs, suggesting inefficient transduction. This was potentially due to the unique transduction characteristics of PFV vectors and their requirement for mitosis.^34^ It is possible that the EGFP-positive cells were those that remained mitotically active in the early postnatal period. This has important implications for therapeutic use of systemically delivered PFV vectors because transduction efficiency may not reach the levels required for gene restoration, even when administered to neonates. But PFV may alternatively possess a useful property in its ability to target cells that remain mitotically active during postnatal development.

PFV vector administration to the neonatal mouse brain gave CNS expression on day 13, which fell 8.2-fold by day 49 post-injection. Direct PFV injection into the CNS has previously been compared with LV vectors in adult rats, where it has been reported that PFV vector transduction was less stable than LVs, potentially because of restrictions on PFV transduction of post-mitotic tissues.^54^

The expression pattern we observed in dorsal brain regions and the spinal cord were most likely due to vector migration through cerebrospinal fluid. The comparatively low level of spinal cord expression on day 13 potentially explains the absence of detectable expression in this region on day 49, given that vector expression in other regions fell substantially across this period.

The fall in expression seen in all tissues between days 13 and 49 could be attributed to a number of factors. Bioluminescent signaling regularly falls after neonatal luciferase gene transfer,^50^, ^55^, ^56^ potentially because of expansion of non-transduced cells, restricting bioluminescence from deeper transduced tissues. Additionally, subgenomic RNA copies are known to be packaged into PFV vector particles during production,^47^ which may have contributed to higher expression levels at the early time point. But our experiments showed that intracranial administration of naked plasmid DNA gave expression that was indistinguishable from uninjected controls, indicating that early expression was primarily derived from the contents of PFV vector particles.

The discrete hippocampal expression profile is an interesting point for more detailed discussion. An intracerebroventricular injection into neonatal mice would normally permit vector spread to distant brain regions. Indeed, we observed broad intracranial diffusion of LV and AAV vectors following neonatal intracranial injection, even when administering these vectors at lower doses than PFVs. It is likely that the biodistribution of LV vectors in the neonatal brain was partly influenced by age-related differences in brain structure, given that LV diffusion was clearly more restricted when administered to adult mouse brains.

However, despite neonatal brain architecture being somewhat permissive to vector diffusion, our data showed that PFV vector expression was highly localized to hippocampal structures, particularly the dentate gyrus. When rationalizing a potential mechanism for this expression pattern, it is important to note that PFV vector transduction has shown dependence on the target cell cycle.^52^ In quiescent cells, PFV capsids pause at the centrosome, and uncoating initiates when cells undergo mitosis.^53^ A study by Caprariello et al.^54^ showed that stable PFV vector expression was detected only in proliferating cells, suggesting that PFV vectors require cell division for stable transduction. This is particularly relevant to our study because hippocampal regions, such as the dentate gyrus, generate neurons postnatally because of proliferation and differentiation of neuronal stem cells.^57^, ^58^ This results in a substantial increase in the size of the dentate gyrus during the first 2 postnatal weeks.^59^, ^60^, ^61^ Thus, there is a possibility that our PFV vector expression pattern relates to unique transduction characteristics, with expression potentially derived from transduction of neuronal progenitors of the dentate gyrus. This would constitute an interesting feature of PFV vector technology because neuronal stem cell pathways could be hijacked and exploited for targeted transgene delivery.

Additionally, our immunostaining images show signs of EGFP expression in microglia and choroid plexus epithelial cells. This observation, along with evidence for PFV transduction of mesenchymal stem cells,^43^ suggests that the postnatal hippocampal expression pattern is attributed to transduction of multiple cell types *in vivo*.

It is often desirable to target discrete brain regions with gene transfer vectors, but we have shown that LV and AAV vectors spread extensively from an injection site and give widespread expression after neonatal injection, meaning that anatomical accuracy is lost without a region-specific promoter.^48^ This constitutes a potential advantage of PFV technology in scenarios requiring expression localized to the hippocampus, such as in the recent correction of an Alzheimer’s disease model by LV-mediated peroxisome proliferator-activated receptor gamma (PPARγ) coactivator 1α gene transfer.^62^ Of course, AAVs may be engineered to confer brain region specificity with customized promoters and regulatory elements, but restrictions regarding AAV packaging capacity mostly hinder inclusion of extensive non-coding sequences. Thus, PFV has the potential advantage of being able to package large transgenes with multiple reporters while retaining region specificity.

## Materials and Methods

### Virus Vector Production

The replication-incompetent prototypic FV clone MD9, containing the EGFP expression cassette, was used as described previously.^36^ A luciferase coding sequence was cloned into the vector using standard molecular cloning techniques. HEK293T cells (6 × 10^6^) were seeded into 10-cm dishes. After 24 hr, cells were transfected using polyethyleneimine. The transfection mix contained 1.5 μg of the PFV vector plasmid pMD9 and 1.5 μg (each) of the packaging plasmids pcoPE, pcoPG4, and pcoPP. 24 hr after transfection, 10 mM sodium butyrate was added for 8 hr to boost cellular transcription. After 48 hr, the supernatant was harvested, filtered (0.45 μm, Millipore), and layered onto 6 mL of a sucrose cushion (20% in medium). The supernatant was centrifuged in a Surespin 630 rotor (Sorvall) at 116,000 × *g* at 4°C for 3 hr before storage at −80°C. PFV-EGFP and PFV-luciferase were used at 1 × 10^10^ and 4 × 10^8^ genome copies/mL, respectively.

Construction of the AAV-CMV EGFP vector (titer, 1 × 10^13^ genome copies/mL) and the LV-EGFP vector (titer, 8.4 × 10^8^ genome copies/mL) have been described previously.^49^, ^50^ The LV-EGFP vector that was administered to adult mice was produced by Oxford Genetics (titer, 8.0 × 10^8^ genome copies/mL). The maps of each vector used in this study are detailed in Figure S6.

### Animal Procedures

The outbred CD1 mice used in this study were supplied by Charles River Laboratories. All animal experiments conducted within this study were in agreement with the United Kingdom Home Office guidelines, approved by the ethical review committee, and followed the institutional guidelines at University College London.

### Vector Administration

For neonatal intracranial injections, vectors were administered using a 33G needle to deliver 2.5 μL of vector (5 μL in the case of LV vectors) into the left lateral ventricle.^63^ On post-natal day 1, non-randomized neonates were subjected to brief hypothermic anesthesia and injected with viral vectors or plasmid DNA via the appropriate route.

For adult intracranial injections, 32-day-old CD1 mice (two females and one male, approximately 20 g body weight) were initially anesthetized with isoflurane and placed in a stereotaxic frame (Kopf Instruments, USA) over a heat mat. Metacam (0.013 mg/kg) and buprenorphine (0.02 mg/kg) were injected via subcutaneous injection. One burr hole was drilled unilaterally at the following coordinates: medium/lateral (ML), 1.00; anteroposterior, −0.2; dorsoventral, −2.00. The coordinates were adjusted in proportion to the distance between the real bregma and lambda over an ML max of 4.00 mm. 4.0 × 10^6^ vector genome copies were injected into the lateral ventricle using a Hamilton syringe at a speed of 250 nL/min.

Plasmid DNA (Figure S3) was injected at a dose of 1.5 μg. Intravenous injections were delivered via the superficial temporal vein in 20 μL volumes. Intra-peritoneal injections were delivered in 200-μL volumes. Subcutaneous injections were delivered in 2.5-μL volumes administered under the skin of the left flank. Experimental groups were blinded during the course of *in vivo* investigations. Each pup received unique identification with a subcutaneous footpad tattoo.

All experiments were carried out under United Kingdom Home Office license PPL 70/8030 and approved by the ethical review committee of University College London.

### Whole-Body and Macroscopic Imaging

Mice injected with the PFV-luciferase vector were subsequently imaged 13 and 49 days after injection by whole-body bioluminescence imaging (IVIS) (Caliper Life Sciences, Hopkinton, MA, USA) as described previously.^55^ Those that received PFV-EGFP were sacrificed on day 11 and examined for direct EGFP expression using a stereoscopic fluorescence microscope (MZ16F, Leica Microsystems, Wetzlar, Germany) as described previously.^56^ Images were captured using a digital microscope camera (DFC420, Leica Microsystems, Milton Keynes, UK) and software (Image Analysis, Leica Microsystems). Mice that received intracerebral injections of double-stranded DNA (dsDNA) plasmid were imaged 5 days and 11 days post-injection, with luciferase activity normalized to the bioluminescent signal produced by replicate 1 of the uninjected group.

### Tissue Preparation

Mice that received neonatal intracranial injections were euthanized by terminal anesthesia 11 days after vector injection before fixing the skinned cranium in 4% paraformaldehyde solution for 24 hr. The brain was carefully excised and fixed for a further 24 hr before transfer to 30% sucrose in 50 mM Tris-buffered saline (TBS).

Adult mice receiving intracranial injections were perfused under terminal anesthesia (sodium pentobarbital) 13 days after vector injection with PBS-heparin (0.8 mg/mL), followed by 4% paraformaldehyde (PFA) in PBS (Santa Cruz Biotechnology). The brains were then removed and left in 4% PFA and PBS overnight at 4°C.

For each sample, 40-μm frozen sections were cut using a Microm HM 430 freezing microtome (Thermo Fisher Scientific, Loughborough, UK).

### Free-Floating Immunohistochemistry

Sections were rinsed with TBS three times for 5 min between each step. Endogenous peroxidase activity was quenched by incubating the sections in 1% hydrogen peroxide in TBS for 30 min. Blocking was carried out for 30 min in a solution of 15% normal goat serum (NGS; Vector Laboratories, Burlingame, CA, USA) in TBS-T (TBS solution containing 0.3% Triton X-100). Sections were incubated overnight at 4°C with rabbit anti-EGFP antibody (1:10,000, ab183734, Abcam, Cambridge, UK), followed by 2 hr with goat anti-rabbit immunoglobulin G (IgG) (Vector Laboratories, PI-1000) at 1:1,000; both antibodies were diluted with 10% NGS in TBS-T. Sections were then incubated for 2 hr in Vectastain ABC (avidin-biotin) solution (ABC, Vector Laboratories, Peterborough, UK) prepared at 1:1000 in TBS 30 min before use. Sections were incubated in the dark in a 0.05% solution of diaminobenzidine (DAB), prepared by dissolving a 10-mg DAB tablet (Sigma, D5905) into 20 mL TBS. After mixing well and filtration through a 0.45-μm syringe filter, 6 μL of 30% hydrogen peroxide was added. Sections were transferred onto gelatin-coated slides in a rostral-caudal order and allowed to dry overnight. They were dehydrated in a series of industrial methylated spirits (IMSs) and placed in HistoClear solution for 20 min before applying coverslips using DPX mounting medium (VWR).

### Immunofluorescence and Scanning Confocal Microscopy

Free-floating brain sections were subjected to fluorescent immunohistochemistry. Antibodies against EGFP and the neuron-specific marker NeuN were used. Sections were initially blocked for 30 min in TBS-T and 15% NGS and then incubated at 4°C overnight with rabbit anti-EGFP (1:4,000) and mouse anti-NeuN (1:500; ABN91, Millipore, Billerica, MA, USA) antibodies made up in TBS-T and 10% NGS. The sections were rinsed three times for 5 min in TBS and then incubated with goat anti-rabbit Alexa 488 (1:1,000, Thermo Fisher Scientific, A-11008) and goat anti-mouse Alexa 546 (1:1,000, Thermo Fisher Scientific, A-11030) for 2 hr. Sections were again rinsed three times in TBS solution and then incubated with DAPI (1:2,000, Invitrogen). Sections were then mounted onto gelatin-coated slides, and coverslips were mounted with Fluoromount G (Southern Biotech, Birmingham, AL, USA). Slides labeled with immunofluorescence were then analyzed, and Z stacks were captured using a laser-scanning confocal microscope (Leica SP5, Leica Microsystems).

### Statistical Analysis

All statistical analyses were carried out using MATLAB 2015a. A two-tailed Welch’s t test was used to compare mean bioluminescence values. This statistical test is robust for datasets without equal variance or sample size. Mouse sample sizes were limited to three or four animals per experimental group for *in vivo* investigations.

## Author Contributions

J.R.C., analysis and review of results and writing of the manuscript; R.K., J.A.D., L.C., T.W., S.M.K.B., S.A., J.N., J.B., R.S., E.L., G.L., F.A., and S.N.W., review and analysis of results and review of the manuscript; R.d.S., D.L., A.R., S.N.W., and S.J.H., planning of experiments, review and analysis of results, and review of the manuscript.

## Conflicts of Interest

The authors declare no competing interests.

## Supplemental Information

Document S1. Figures S1–S6

Document S2. Article plus Supplemental Information
