# Supplementary material for: Foamy Virus Vectors Transduce Visceral Organs and Hippocampal Structures following In Vivo Delivery to Neonatal Mice
Source: Mol Ther Nucleic Acids. 2018 Aug 3;12:626–34. doi: 10.1016/j.omtn.2018.07.006 (PMC6082918; doi:10.1016/j.omtn.2018.07.006)
Supplement: Document S1. Figures S1–S6 [file mmc1.pdf]

OMTN, Volume *12*

Supplemental Information

Foamy Virus Vectors Transduce Visceral Organs and Hippocampal Structures following *In Vivo* Delivery to Neonatal Mice

John R. Counsell, Rajvinder Karda, Juan Antinao Diaz, Louise Carey, Tatiana Wiktorowicz, Suzanne M.K. Buckley, Shima Ameri, Joanne Ng, Julien Baruteau, Filipa Almeida, Rohan de Silva, Roberto Simone, Eleonora Lugarà, Gabriele Lignani, Dirk Lindemann, Axel Rethwilm, Ahad A. Rahim, Simon N. Waddington, and Steven J. Howe

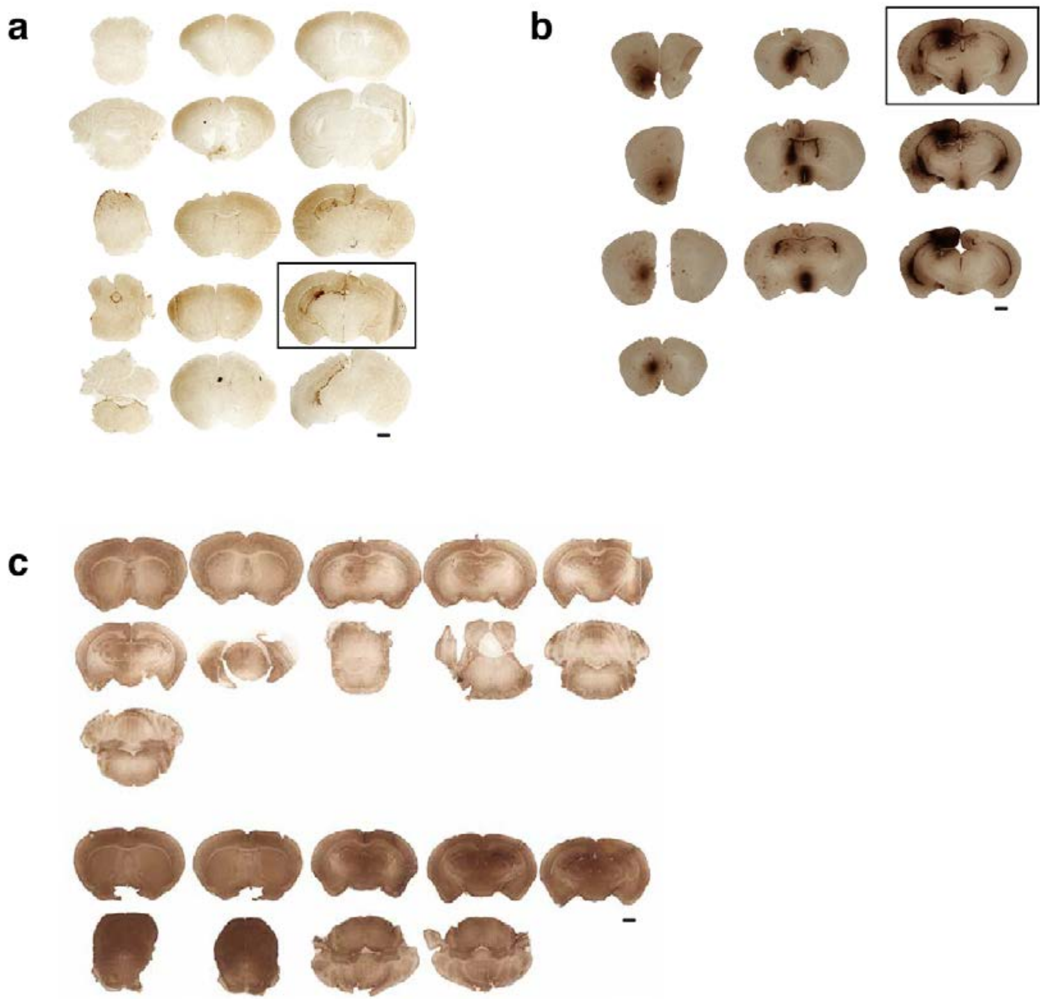

Supplementary Figure S1. Brain eGFP expression and tropism of PFV-CMV-eGFP expression 11 days after intracranial vector injection *versus* AAV vector tropism. **a.** Sagittal slices of brains injected with PFV-CMV-eGFP show eGFP immunostaining localized to the dentate gyrus. **b.** LV-eGFP-treated sections show more widespread staining throughout the brain, with diffused spread from the injected ventricle. **c.** Conversely, delivery of AAV-eGFP shows unrestricted spread of vector expression throughout all brain slices without any site-specific tropism. Black boxes highlight the sections that have been presented in Figure 4. Scale bars = 5μm.

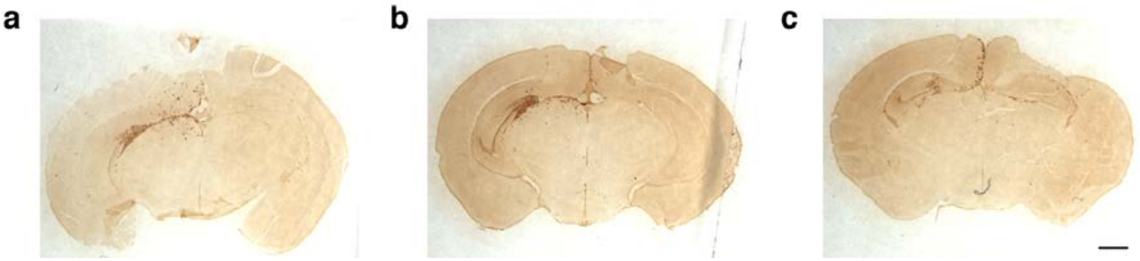

Supplementary Figure S2: Coronal slices of the three mouse brains injected with PFV-eGFP. Replicates **a**, **b** and **c** show hippocampal staining after lateral ventricle administration of PFV-eGFP. Replicate **b** is the sample relating to images in Figure 4 of the manuscript and Supplementary Figure S1. Scale bar = 5µm.

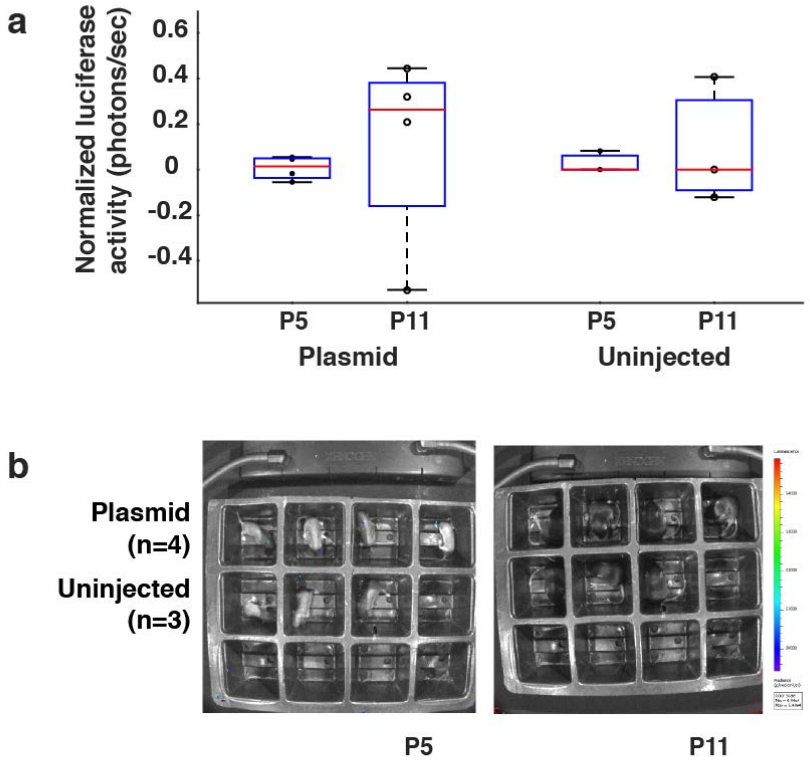

<u>Supplementary Figure S3: Plasmid DNA does not appear to be a major factor in the expression detected in PFV-Luciferase-injected animals at early time-points.</u> Plasmid DNA containing luciferase under the transcriptional control of the spleen-focus-forming virus (SFFV) promoter (pLNT-SFFV-JDG) was delivered to neonatal mice by unilateral intracranial injection at P1. **a.** Quantification of whole body bioluminescence imaging taken from plasmid-injected and uninjected mice at days 5 and 11 post-injection shows no discernable difference between the two groups. The mean luciferase activity detected in each animal is plotted as an individual dot-plot (black dots = day 5 values; white circles = day 11 values) with overlaid boxplots representing 75% confidence intervals of the dotplot distribution (blue boxes) and median lines (red lines). In each case, the luciferase activity is normalized to the bioluminescent signal produced by replicate-1 of the uninjected group. **b**. Representative images taken during IVIS imaging show that bioluminescent signal is minimal at both time-points.

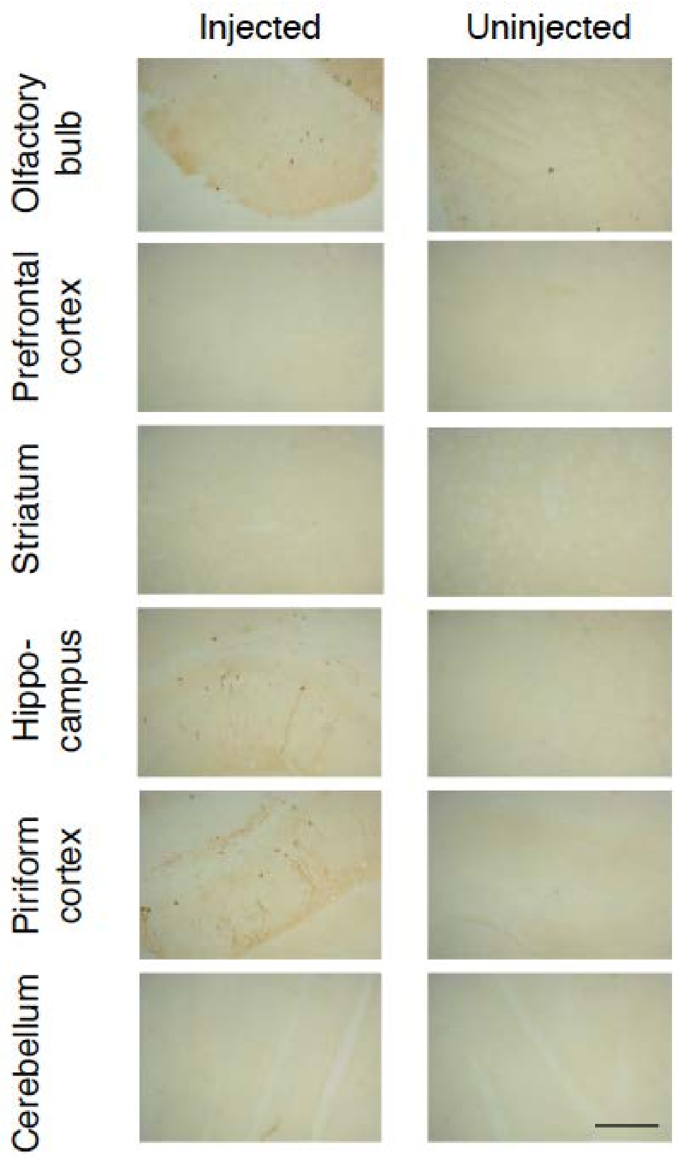

Supplementary Figure S4: LV-eGFP administration to adult mouse brain gives restricted expression.
A dose of 4 x 10^6 LV-eGFP vector genome copies was injected into an adult mouse brain and analyzed for eGFP expression 13 days later. Coronal brain sections (left panels) showed sparse eGFP immunostaining localized to the hippocampus, piriform cortex and olfactory bulb, but absent from other regions. Scale bar = 100µm.

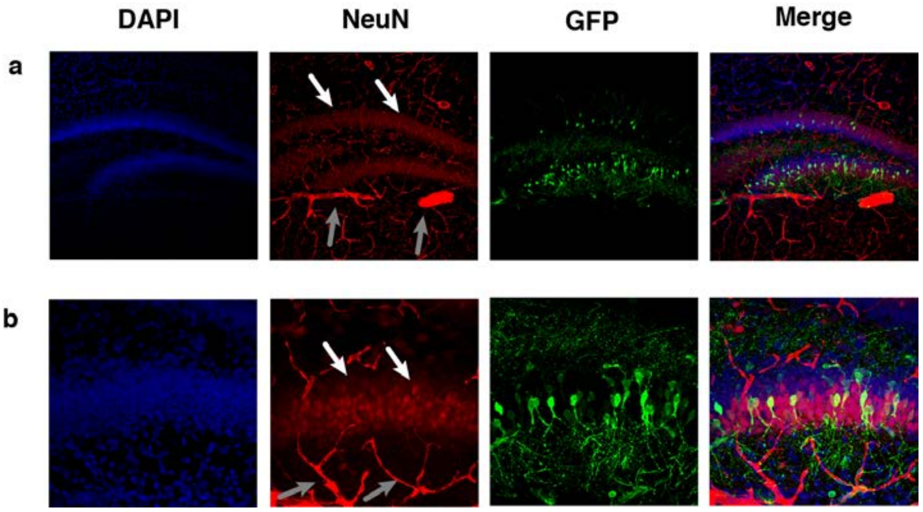

<u>Figure S5: Co-localisation of eGFP expression and NeuN expression in the dentate gyrus of PFV-CMV-eGFP treated mice.</u> Brain sections from injected mice were used to determine the cell types expressing GFP after neonatal intracranial injections of PFV-CMV-eGFP. Images show staning patterns for GFP (green), NeuN (red) in the dentate gyrus, or DAPI nuclear staining (blue). **a.** A representative brain section of the hippocampus taken at 20x magnification. **b.** The same image taken at 40x magnification for closer inspection of hippocampal architecture.

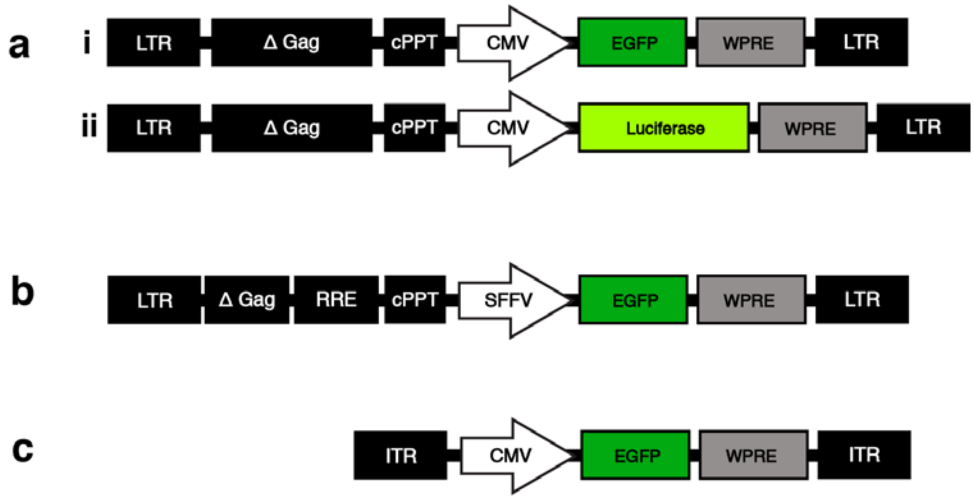

Supplementary Figure S6: Maps of vector genomes employed in this study. **a.** PFV vector genomes were packaged with either (i) EGFP or (ii) luciferase, driven by the CMV promoter in each case. **b.** LV-eGFP was packaged with eGFP transgene driven by the spleen-focus forming virus (SFFV) promoter. **c.** AAV-eGFP was packaged with an eGFP transgene driven by the CMV promoter.
